# Utility of brGDGTs as temperature and precipitation proxies in subtropical China

**DOI:** 10.1038/s41598-017-17964-0

**Published:** 2018-01-09

**Authors:** Mengyuan Wang, Yongqiang Zong, Zhuo Zheng, Meiling Man, Jianfang Hu, Liping Tian

**Affiliations:** 1 School of Earth Science and Geological Engineering, Sun Yat-Sen University, Guangzhou, 510275 China; 2 Department of Earth Sciences, The University of Hong Kong, Hong Kong SAR, China; 3 State Key Laboratory of Organic Geochemistry, Guangzhou Institute of Geochemistry, Chinese Academy of Sciences, Guangzhou, 510640 China

## Abstract

Bacterial branched glycerol dialkyl glycerol tetraethers (brGDGTs) have been successfully used as quantitative climate proxies for reconstructing annual mean air temperature (MAT) and soil pH from sediments. However, reconstructions derived from brGDGTs in regions with diverse hydrological and atmospheric conditions require further refinement. In this study, we investigated the suitability of brGDGTs as temperature and precipitation proxies from surface soils on opposite slopes of Mount Fanjing in subtropical China. The results show a clear altitudinal lapse rate of MBT′_5ME_-derived MAT and between-slope differences in MAT at given altitude. Moreover, the MBT′_5ME_-derived MAT values are more strongly related to the MAT from March to November than that of the whole year. A turning point is also observed from the measured pH and CBT′-derived pH gradients at altitude of 1400 m~1500 m, in accordance with the fog horizon, where precipitation reaches the maximum value. The findings prove that brGDGTs from soil transects can be used as indicators for reconstructions of climate parameters from subtropical regions of China.

## Introduction

Glycerol dialkyl glycerol tetraethers (GDGTs) are membrane lipids that are ubiquitous in diverse environments^1^. They include isoprenoid GDGTs (isoGDGTs) produced by Archaea and branched GDGTs (brGDGTs), originated from unknown heterotrophic bacteria, some of which might belong to the phylum Acidobacteria^2–6^. IsoGDGTs dominate in marine^7–10^ and some lacustrine^11^ environments. The relative abundance of specific isoGDGTs forms the basis of the TEX_86_ index (tetraether index of tetraethers consisting of 86 carbons^7^). It has been shown that TEX_86_ index is correlated with sea surface temperature (SST^7,12,13^) and lake surface temperature^11,14^. Dry and alkaline soils in China also contain substantial amounts of isoGDGTs^15,16^. However, BrGDGTs are generally abundant in soils and form two useful indicators: the methylation index of branched tetraethers (MBT) and cyclisation ratio of branched tetraethers (CBT). The CBT of brGDGTs is mainly controlled by soil pH or water availability^16,17^, whilst the MBT seems to be strongly governed by annual mean air temperature (MAT) and soil pH^18,19^. Thus, brGDGTs in soils^18,20^, peat^21–23^, lakes^14^, estuary^24^ and marine sediments^25^ have been widely used in climate parameters reconstructions in the past few years.

The CBT and a combination of the CBT and MBT (CBT-MBT) can be respectively used for soil pH and MAT reconstruction^18,26^. The utility of CBT and MBT values as pH and MAT proxies has been tested using surface soil samples from transects in numerous locations, e.g. Mt. Kilimanjaro^27^ and Mt. Rungwe in Africa^28^ in Tanzania; Southern Alps^29^ and Eastern Cordillera of Colombia^30^; Mt. Gongga^31^, southeastern slope of Tibetan plateau^32^, Mt. Xiangpi^33^, Mt. Shennongjia in China^34^, and Mt. Meghalaya in India^35^. CBT and MBT proxies have been applied to a number of slopes for examining their accuracy along environmental gradients^31^.

**Figure 1:**
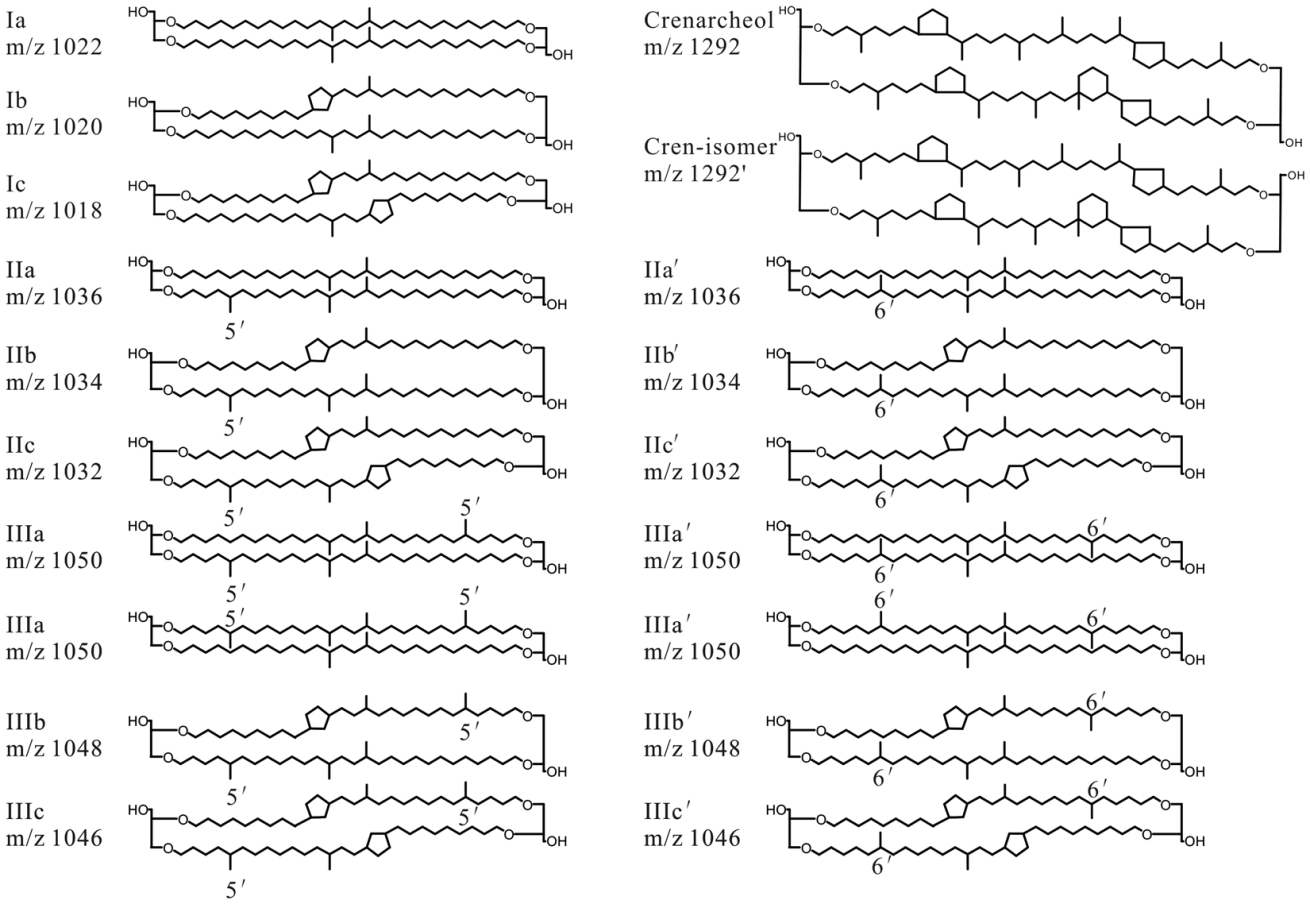
Chemical structures of branched GDGTs (I-III) and crenarchaeol (IV).

Furthermore, about 10 years ago Weijers^18^ published calibrations for deriving MAT from MBT-CBT data and for deriving soil pH from CBT data obtained from samples collected from 134 globally distributed sites in more than 90 regions. Subsequently, Peterse^26^ revised the calibrations by extending the dataset to surface soils of 278 globally distributed soils. Tests of the new MBT′-CBT function showed that it provides a better agreement with MAT and pH measurements. However, unsurprisingly, local and regional soil data provide more accurate calibrations than the global dataset for local and regional climate reconstructions^27^. For example, the analyses of more than 100 soil samples from sites in northern and central China by Yang^36^ have provided more appropriate calibrations for arid and semi-arid soils in China. Recently, De Jonge^37^ proposed MBT′_5ME_ and CBT′ which were defined with the separated 5-methyl and 6-methyl brGDGTs based on an improved liquid chromatography method. The 6-methyl brGDGTs are denoted by an accent after the roman numerals for their corresponding 5-methyl isomers (Fig. 1). The newly exhibited proxies showed that MBT′_5ME_ was no longer related to soil pH, and its correlation with MAT was improved. Meanwhile, CBT′ was recommended for reconstructing soil pH because the Root Mean Squared Errors (RMSE) of their calibrations was reduced from 0.8 to 0.5. Yang^34^ reported a series of transect data from Mt. Shennongjia (northern subtropical China) and proved that MBT′_5ME_ appeared more significantly correlated with MAT than MBT′ was. However, the information on MBT′_5ME_ and CBT′ profiles of soils in subtropical China, where the soils are more diverse and the climate is warm and rainy^33^, are still lacking. It is likely that analyses of the separated 5-methyl brGDGTs in surface soils of subtropical China can provide valuable new information for climate reconstructions.

**Figure 2:**
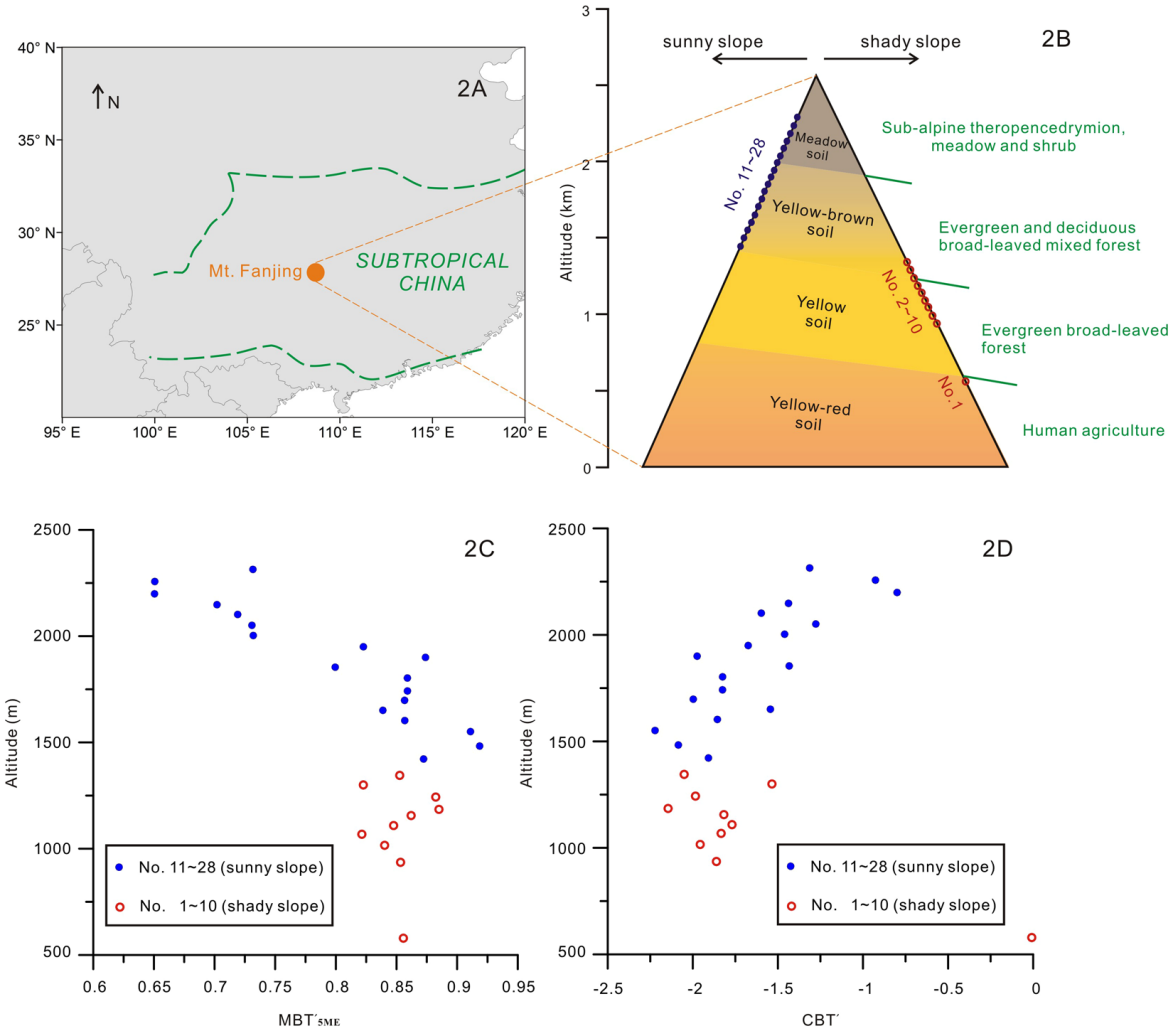
(**A**) Location of Mt. Fanjing and the region of subtropical China. Map was produced using ArcGIS 10.5 (http://esrichina.hk/); (**B**) Simplified slopes, vertical vegetation zones, and soil sampling sites (1 to 10 on the shady slope, and 11 to 28 on the sunny slope); (**C**) A plot between MBT′_5ME_ and altitude; (**D**) A plot between CBT′ and altitude.

To further improve understanding of the correlations between the two new brGDGT proxies (CBT′ and MBT′_5ME_), and both hydrological and temperature climatic parameters, we have investigated brGDGTs in surface soils along two altitudinal gradients from 579 m to 2314 m on Mount Fanjing of the Wuling Range, Guizhou Province, subtropical China (Fig. 2). This region of central-south China area is strongly influenced by the Summer Monsoon and receives more than 1100 mm precipitation per year. On Mount Fanjing, there is strong vertical zonation in its vegetation, climate and soils. Thus, we assume that an analysis of altitudinal changes in brGDGTs from this mountain can enhance understanding of their potential utility as palaeo-climate proxies, especially for precipitation and temperature, in subtropical China.

## Results

In order to examine the suitability of brGDGT-derived proxies, local temperature, precipitation, soil pH value and water content are measured or estimated for each sampling location. The results (Supplementary Table 1) show some important patterns. (1) The 9-warm-month (9-month) MAT decreases linearly from about 19.5 °C at 500 m to 15.5 °C at 1400 m of the shady slope, and decreases linearly from 19.5 °C at 1400 m to 14.0 °C at 2400 m of the sunny slope. In other words, there is a constant difference of 4.0 °C between the two slopes. (2) The annual mean precipitation increases linearly from about 1500 mm at 500 m to 2600 mm at 1400 m of the shady slope. On the sunny slope, annual mean precipitation increases linearly from 2550 mm at 1400 m to 3000 mm at 1700 m and decreases to 2000 mm at 2400 m. The difference of precipitation between the two slopes is very small, and the highest precipitation is recorded at c. 1700 m. (3) Soil pH values change gradually from 5.0 at 500 m to 4.2 at 1400 m of the shady slope. On the sunny slope, however, a reverse trend is observed as the values change from about 4.0 at 1400 m gradually to 5.7 at 2300 m, except the sample from 2314 m that has a pH value of 4.0. In other words, the relationship between pH values and altitude is largely similar to that between precipitation and altitude. (4) Water content (%) of the soil samples varies between 2% and 90%, and show no particular pattern with altitude.

brGDGTs and isoGDGTs are detected in all samples and presented in the supplementary Table 1. For all the samples, brGDGTs are more abundant than isoGDGTs, and their average fractional abundances are 62.23%~98.42% and 1.58%~37.77% respectively. Within brGDGTs, compounds Ia and IIa (Fig. 1) are the main components (20%~90% and 5%~30%, respectively), compounds IIIc and IIIb brGDGTs are barely detectable in most samples, and fractional abundance of compounds IIc and IIb are also relatively low or just about detectable. This distribution pattern is similar to Peterse^26^.

The values of MBT′_5ME_ vary from 0.60 to 0.95 (Fig. 2C). Data from the shady slope between 500 m and 1400 m show no obvious trend between MBT′_5ME_ values and altitude. On the sunny slope between 1400 m and 2400 m, however, a linear trend can be observed. The values of CBT′ fall into the range between −2.2 and −0.8, except the sample collected at 579 m of altitude that has a value close to 0. Similar to MBT′_5ME_, the CBT′ values from 900 m to 1400 m on the shady slope show no obvious trend with altitude, but a clearer linear trend with altitude is apparent for the CBT′ values from 1400 m to 2400 m on the sunny slope (Fig. 2D).

## Discussion

### MBT′_5ME_-derived MAT

**Figure 3:**
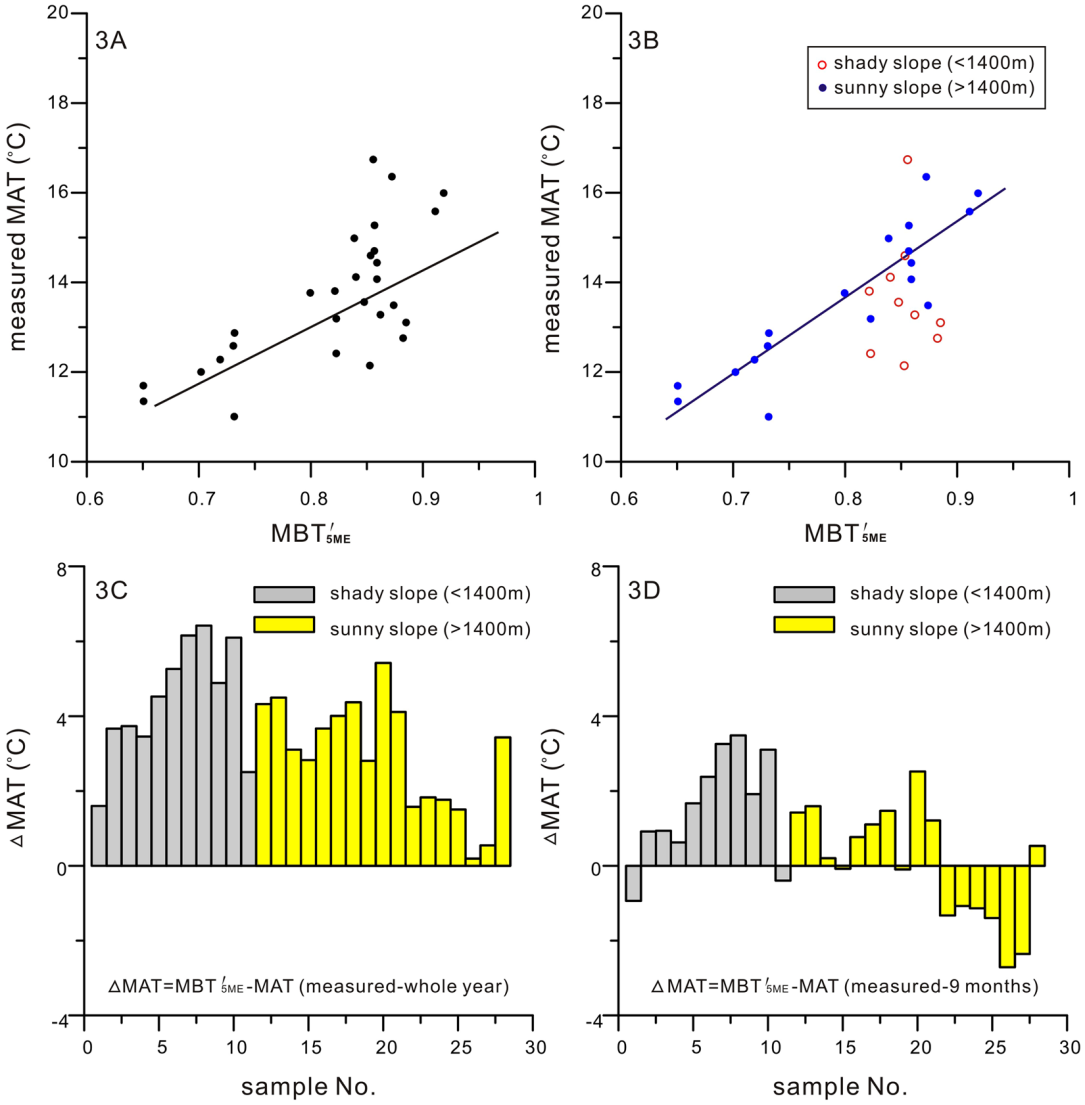
(**A**) A plot between MBT′_5ME_ and measured MAT (°C); (**B**) The same plot as A, but the samples are separated by different mountain slopes, and the correlation is only based on samples of the sunny slope (solid dots). (**C**) MAT (°C) differences between MBT′_5ME_-MAT and measured MAT (°C) of the whole year; (**D**) MAT (°C) differences between MBT′_5ME_-MAT and measured MAT of 9 months (March to November).

The correlation between MBT′_5ME_ and measured MAT for all the 28 samples exhibits a relatively low correlation coefficient (*r* ^2^ = 0.51, *p* < 0.0001, *n* = 28) (Fig. 3A), which is slightly lower than the one proposed by De Jonge^37^ (*r* ^2^ = 0.66, *n* = 222). When these samples are separated into two groups, the sunny slope and the shady slope, the correlation coefficients are very different (Fig. 3B). Samples from the sunny slope show a much stronger, linear correlation between measured MAT and MBT′_5ME_, and the correlation yields a high coefficient (*r* ^2^ = 0.74, *p* < 0.0001, *n* = 18) and a small RMSE ( ± 0.82 °C). The correlation (*r* ^2^) and precision (RMSE) from this local data set are significantly better than that of the global data set reported by De Jonge^37^, and it suggests that local or regional data sets offer greater accuracy in calibrations than the global dataset when reconstructing local/regional climate parameters^26,27^. On the other hand, the samples’ MBT′_5ME_ of the shady slope show almost no correlation with measured MAT. One reason for this poor correlation in the shady slope could be that the sampling sites are under full coverage of evergreen broad-leaved forest, and as a result temperature difference in the soil is less sensitive to air temperature. On the sunny slope, the soil is less densely covered by vegetation and much more exposed to sun light. Thus soil MBT′_5ME_ from the sunny slope show strong correlation with measured MAT.

As stated above, soil MBT′_5ME_ is well correlated with measured MAT, particularly in the sunny slope of Mt. Fanjing. To examine the applicability of the soil MBT′_5ME_ for reconstructions, the calibration function proposed by De Jonge^37^ is used to derive the MBT′_5ME_-based MAT for samples collected from the study area, and produced ΔMAT with subtracting the measured MAT and the 9-month MAT by the MBT′_5ME_-based MAT. The results show that the MBT′_5ME_-based MATs are consistently higher than the measured MATs (Fig. 3C), but they are much closer to the 9-month MATs (Fig. 3D). In more details, the calibrated MBT′_5ME_-MATs are about 1~7 °C higher than the measured MATs. On the other hand, the calibrated MBT′_5ME_-MATs are only in small variations with the 9-month MATs. It is likely that the measured annual air temperature is the annual average value, rather than the average temperature above the threshold for biological activity of the brGDGT-producing organisms. As previously reported, brGDGT-producing bacteria are more active during spring, summer and autumn than during the rest of the year^23,38^, thus the calibrated MBT′_5ME_-MATs may be more consistent with warmer seasonal temperatures^32,38^. Moreover, as 5.5 °C is the lowest optimal base for experimentally determining the lifecycle of the plant or insect^39^, e.g. Growing Day Degrees >5.5 °C (GDD 5.5; the cumulative daily mean temperature above 5.5 °C) may be a better indicator of the bacterial activities. Temperature data from Mt. Fanjing meteorological station confirms that most of the temperatures lower than 5.5 °C is recorded in the months of January, February and December. In other words, the 9-month MATs (exclude Jan., Feb. and Dec.) are more appropriate for comparison (Fig. 3D). Since the RMSE of the global MBT′_5ME_-MAT calibration function is 4.8 °C^37^, the ± 4 °C temperature differences (Fig. 3D) fall within the error range. This analysis, therefore, confirm that the MBT′_5ME_ is applicable for reconstructing Mt. Fanjing 9-month MAT, and it agrees with the findings that brGDGTs production is seasonally biased^32,38^. What’s more, the uncertainty of the global soil calibration (RMSE =  ± 4.8 °C) is also one of the reasons attributed to the temperature differences between MBT′_5ME_ derived and measured MAT values.

**Figure 4:**
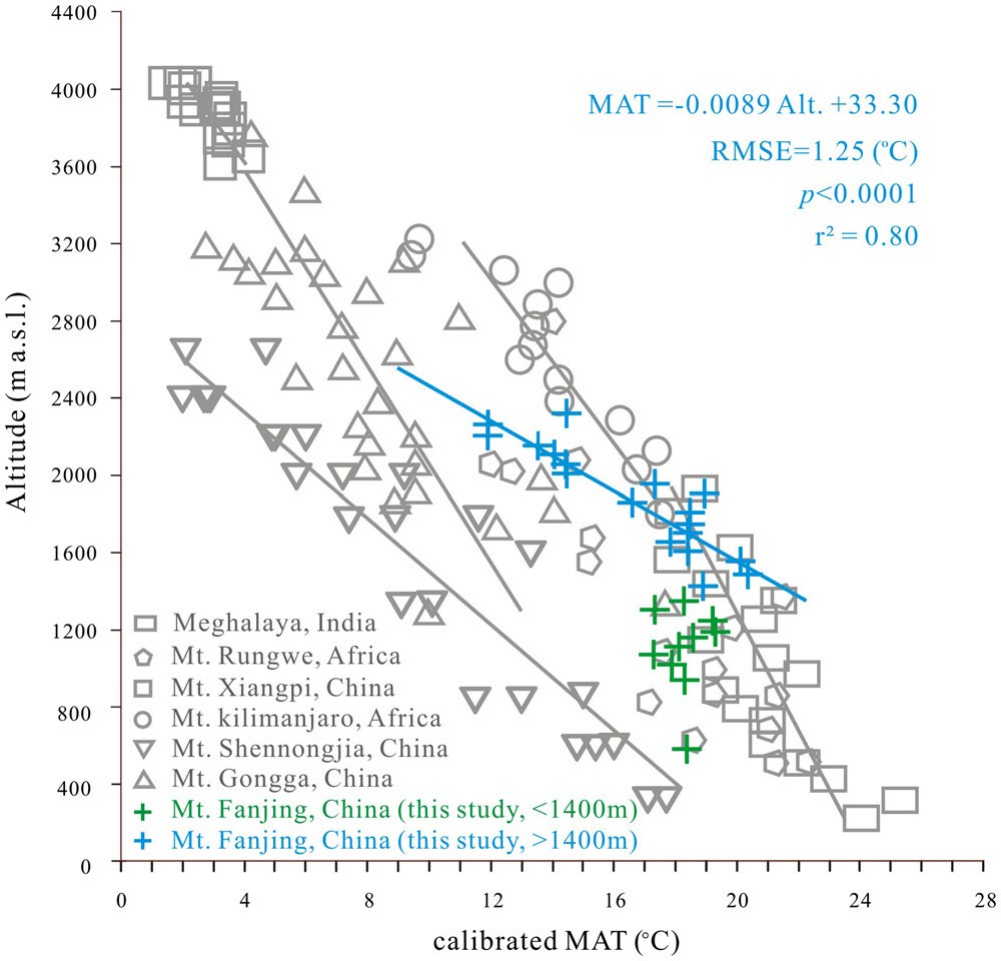
brGDGT-based MATs along studied altitudinal gradients on Mt. Fanjing, China, calculated according to the calibration from Peterse^26^ and De Jonge^37^: green crosses are samples from the shady slope and blue crosses are samples from the sunny slope. Samples from the sunny slope show a lapse rate of 0.89 °C/100 m (r^2^ = 0.80 and RMSE = 1.25 °C; MBT′_5ME_-MAT), compared with Meghalaya^35^, India (rectangle; r^2^ = 0.66; MBT′-MAT), Mt. Gongga^31^, China (triangle; r^2^ = 0.56; MBT′-MAT), Mt. Kilimanjaro^27^, Tanzania (circle; r^2^ = 0.77; MBT′-MAT), Mt. Xiangpi^33^, China (square; r^2^ = 0.60; MBT′-MAT), Mt. Rungwe^28^, Tanzania (pentagon; r^2^ = 0.74; MBT′-MAT) and Mt. Shennongjia^34^, China (inverted triangle; r^2^ = 0.90; MBT′_5ME_-MAT).

The brGDGT-derived MAT decreased linearly with altitude on the sunny slope (Fig. 4), as previously observed at many other locations^27,28,31,33,35^. Values derived from the calibration function presented by De Jonge^37^ lapsed linearly at 0.89 °C/100 m of altitudes on the sunny slope of Mt. Fanjing (r^2^ = 0.80). The lapse rate is slightly higher than those obtained from regional meteorological observations and reconstructions from nearby regions, e.g. 0.59 °C/100 m at Mt. Gongga^31^ (southwest China, MBT′-MAT), 0.63 °C/100 m at Mt. Xiangpi^33^ (northwest China, MBT′-MAT) and 0.63 °C/100 m Mt. Shennongjia^34^ (northern subtropical China, MBT′_5ME_-MAT) (Fig. 4). On the other hand, the MBT′_5ME_-MATs from the shady slope show no correlation with altitude (Fig. 4). The reason for it needs to be investigated in the future.

### CBT′-derived pH

**Figure 5:**
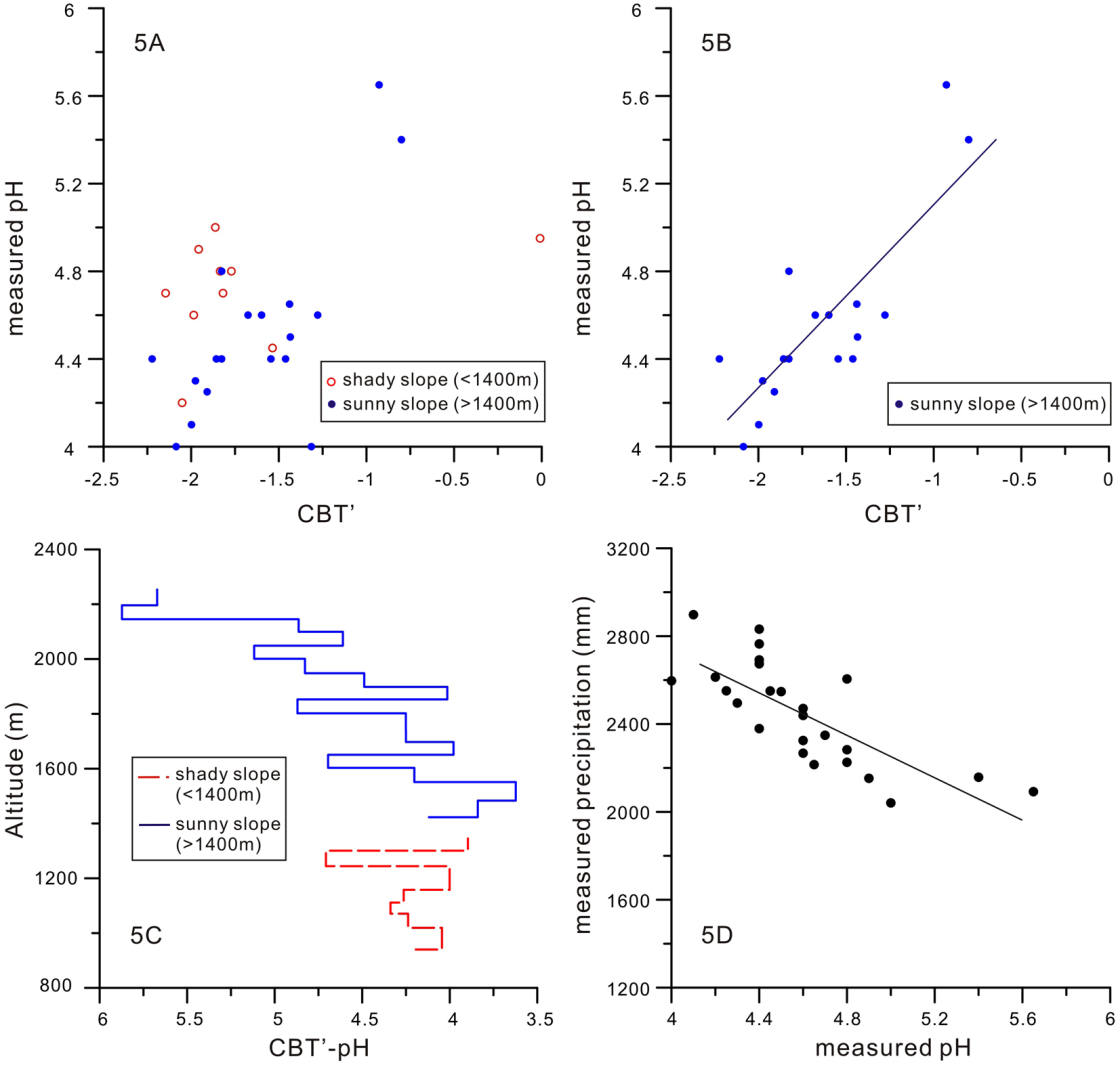
(**A**) A plot of CBT′ and measured pH; (**B**) A plot of CBT′ and measured pH for samples of sunny slope only; (**C**) CBT′- derived pH variation along the altitude of Mt. Fanjing; (**D**) Correlation between measured pH and precipitation (mm) for samples of both sunny and shady slopes.

To evaluate the potential utility of CBT′-derived pH as a soil pH proxy, it is essential to determine the relationship between CBT′ and measured pH. As shown in the scatter plot (Fig. 5A), the CBT′ values from both slopes is only weakly correlated to the measured soil pH (*r* ^2^ = 0.39), which is generally in support of the global calibration^37^. If only the samples from the sunny slope are used, the correlation coefficient is improved to 0.69 (Fig. 5B). The RMSE of this correlation is 0.24, which is lower than that of the global calibration (0.52). Therefore, in both cases, the precision of pH calibration using local data set increases substantially in comparison with the global one.

With such confirmation, CBT′-derived pH is calculated using the global CBT′-pH calibration^37^. The results confirm that most of the differences between the measured and the CBT′-derived pH are within the global calibration RMSE (0.52). Excluding three samples from which the CBT′-derived pH is far beyond the RMSE range, the resulted CBT′-derived pH along altitude of Mt. Fanjing is presented in Fig. 5C. It shows that the lowest pH appears at the altitude around 1400 m~1500 m. Upwards from this altitude, the CBT′-derived pH increases gradually. This trend follows the precipitation. The lower the precipitation is, the lower level of soil humification is recorded, probably leading to lower organic acid production rates and higher pH values^40^, which is observed by the close correlation (*r* ^2^ = 0.58) between measured pH and precipitation values (Fig. 5D). Thus, the CBT′-derived pH can be used to infer precipitation. In summary, the comparison between CBT′-derived pH and measured pH proves that brGDGTs can be a potential precipitation proxy along an altitudinal gradient as confirmed in the case of Mt. Fanjing.

## Conclusions

This study confirms that brGDGTs from soils can be used as indicators to reconstruct climate parameters on Mt. Fanjing, subtropical China. Firstly, MBT′_5ME_ is found well correlated with measured MAT at Mt. Fanjing, especially for soil samples from the sunny slope at the altitude above 1400 m. The results show that the RMSE of this local data set is smaller than the global calibration set; the correlation coefficient between MBT′_5ME_–derived MAT and the measured MAT is 0.80, higher than the global calibration set; the lapse rate (0.89 °C/100 m) is larger than some other studies. It is also observed that the MBT′_5ME_-derived MAT values tend to be 1 to 7 °C higher than the measured MAT values on both slopes, but much closer to the 9-month MAT (i.e. excluding three winter months), confirming the importance of seasonal bacterial productivity. Besides, the uncertainty of the global soil calibration is also one of the reasons attributed to the temperature differences between MBT′_5ME_ derived and measured MAT values. However, soil samples from the shady slope between 500 m and 1400 m of altitude show weak correlation with measured MAT, possibly due to the fact that dense coverage of broadleaved forest may have provided a thermal protection to the soil. Thus, mountain slopes should be taken into consideration when sampling soils from an altitudinal transect. Secondly, the difference in values between CBT′-derived soil pH and measured pH is mostly within the RMSE of the global calibration set. CBT′-derived pH is not linearly correlated with altitude of Mt. Fanjing, but correlated well with measured pH. Since both the measured pH and CBT′-derived soil pH are strongly correlated with precipitation, the CBT′-derived pH can be used for precipitation reconstructions.

## Methods

### Study site

Mt. Fanjing (27°47′50′′−28°1′30′′N, 108°45′55′′−108°48′30′′E) is situated in the transitional zone between the Yunnan-Guizhou Plateau and Xiangxi mountain ranges, in the northeastern part of Guizhou Province of China, close to the junction of Yinjiang, Song Tao and Jiangkou counties^41,42^. The mountain is 27 km long from south to north and 21 km wide from east to west, covering an area of ca. 567 km^2^. It has steep slopes and several peaks, with heights ranging from 500 m (Panxikou in the east) to 2572 m (Mt. Fenghuang). The northeast side is shady and windward, whilst the southwest side is sunny and warm. Being located in the center of subtropical China (Fig. 2A), it is under the typically mid-subtropical humid monsoon mountain climate. The area is the foggiest in Guizhou province, with up to 49 foggy days per year^43^.

Dense and well-preserved forest covers more than 80% of the mountain, but there is a clear vertical zonation on every side (Fig. 2B). The east slope is cooler than the west slope, because it not only receives less insolation but also lies in the direct path of cold air flow coming into Guizhou Province from the north^44^. Generally, the vegetation on the southwest/northwest slope is agricultural, yellow-red soil at altitudes below 800 m, evergreen broadleaved forest with yellow soil from 800 to 1400 m, deciduous broadleaved mixed forest with yellow-brown soil from 1400 to 2000 m, and alpine meadow with brown meadow soil above 2000 m. The associated vegetation zones on the northeast/southeast slope extend to 600 m, from 600 to 1250 m, from 1250 to 1900 m and above 1900 m, respectively^44,45^.

According to the measurements of Mt. Fanjing meteorological station (on the northern slope at 2255 m altitude), most precipitation falls from April to October, the mean annual precipitation (based on reference data for 2012–2013) is 2480 mm, and mean annual humidity exceeds 90%^46^. Highest precipitation is recorded at 1700 m of altitude according to Zhong^44^. Annual air temperature decreases upwards by 0.5~0.56 °C and 0.6 °C per 100 m on the mountain slope according to Zhong^44^ and Xie^46^, respectively. Air temperatures may reach 30 °C in July and August and fall below 0 °C in December, January and February. The mean annual temperature (MAT) at 2255 m of Mt. Fanjing is 8.4 °C.

### Sample collection

In total, 28 samples of surface soil (0–5 cm) were collected along altitudinal gradients between 579 m and 2314 m in 2015 (Supplementary Table 1, Fig. 2): ten (at altitudes from 579 to 1345 m) along a transect on the northeast slope, and eighteen (at altitudes from 1422 m to 2314 m) along a transect on the southwest slope (Fig. 2).

### Soil pH and soil water content measurements

Following Weijers^18^, triplicate portions of each surface soil sample were mixed with ultra-pure water at a ratio of 1:2.5 (g/ml); the pH value of the supernatant was measured using a pH meter (EZDO PH7200 waterproof pen) with a precision of ± 0.01, and the mean (standard deviation, ± 0.04) was recorded as the sample’s pH value. Soil water content was calculated by measuring the loss weigh of water from the net weight of wet soils before and after the samples being put into an oven set at the temperature of 105 °C.

### Environmental parameters

The climate information for each sampling site was obtained from the Worldclim dataset at a spatial resolution of 2~5 minutes. The software used for data extraction is DIVA-GIS. The MAT and annual mean precipitation (MAP) data are the average values for 1950~2000. The 9 months MAT data was obtained from mean air temperature ranging from March to November. All the collected data are calculated and adjusted according to sample altitude, the slopes^44^ and the nearest meteorological station recorded data (http://cdc.cma.gov.cn/).

### Lipid extraction and GDGT analysis

Aliquots of the soil samples were prepared for GDGT analysis by freeze-dried at −18 °C in a refrigerator. The soils were ground into less than 200 mesh size, and about 5.0 gram of the subsamples were spiked with a known amount of C_46_ GDGT internal standard (IS)^47^. An organic solvent (9:1 dichloromethane: methanol) was added to each sample to extract organic compounds using ultrasonic extraction at least three times. *n*-Hexane was added to obtain the neutral extracts (three times). The neutral extracts were then purified and separated by silica-gel chromatography using hexane/DCM (9:1) and DCM/methanol (1:1) as subsequent eluents to separate into non-polar and polar fractions. The polar fraction containing the GDGTs was dried under nitrogen gas and then re-dissolved in hexane/isopropanol (99:1, v/v). The resulting samples were passed through a 0.45 µm polytetrafluoroethene filter before analysis.

The GDGT were analyzed at the Institute of Earth Environment, Chinese Academy of Sciences by HPLC-atmospheric pressure chemical ionization-mass spectrometry (HPLC-APCI-MS), performed with a Shimadzu LC-MS 8030. BrGDGTs were separated with two coupled Inertsil SIL-100A silica columns (each 250 mm × 4.6 mm, 3 μm; GL sciences Inc.) at 40 °C using isopropanol and *n*-hexane as elutes for pump A and pump B, respectively (modified from De Jonge^37^ and Yang^34^). Selected ion monitoring (SIM) was used to target specific [M + H]^+^, including those for the brGDGTs ([M + H]^+^ 1050 IIIa III′a, 1048 IIIb III′b, 1046 IIIc III′c, 1036 IIa II′a, 1034 IIb II′b, 1032 IIc II′c, 1022 Ia, 1020 Ib and 1018 Ic). The relative abundances of individual brGDGTs were calculated according to the integrated peak areas. MBT′_5ME_, CBT′ were calculated based on the specific brGDGT group (5- and 6-methyl brGDGTs), where MBT′_5ME_ = (Ia + Ib + Ic)/(Ia + Ib + Ic + IIa + IIb + IIc + IIIa) and CBT′ = ^10^log[(Ic + IIa′ + IIb′ + IIc′ + IIIa′ + IIIb′ + IIIc′)/(Ia + IIa + IIIa)]^37^. Replicate HPLC/APCI-MS analysis of samples showed the reproducibility of MBT′_5ME_ and CBT′ (Supplementary Table 1) brGDGTs to be ± 0.0019 and ± 0.018, respectively. The transfer functions of MBT′_5ME_-MAT and CBT′-pH are: MAT = −8.57 + 31.45 × MBT′_5ME_ (r^2^ = 0.66, RMSE = 4.8 °C) and pH = 7.15 + 1.59 × CBT′ (r^2^ = 0.85, RMSE = 0.52)^37^.

### Data Availability

The datasets generated during and/or analysed during the current study are available in the supplementary Table 1.

## Electronic supplementary material

Supplementary Information 
